# 

**Published:** 1896-05

**Authors:** 


### International Dental Journal

A MONTHLY PERIODICAL

DEVOTED TO

### DENTAL AND ORAL SCIENCE.

JAMES TRUMAN, D.D.S., EDITOR.

GEORGE W. WARREN, D.D.S., ASSISTANT EDITOR.

EDITORIAL OFFICE, 4505 CHESTER AVENUE, PHILADELPHIA, PA.

☞SUBSCRIPTIONS AND COMMUNICATIONS RELATING TO THE BUSINESS MANAGEMENT SHOULD BE ADDRESSED TO THE

PUBLICATION OFFICE, 716 FILBERT STREET, PHILADELPHIA, PA.

PUBLISHED BY THE

INTERNATIONAL DENTAL PUBLICATION COMPANY,

51 WEST THIRTY-SEVENTH STREET, NEW YORK CITY,

—AND—

716 FILBERT STREET, PHILADELPHIA.

HARRY T. PEARCE, ADVERTISING MANAGER, 1914-16 CHERRY ST., PHILADELPHIA, PA.

Entered at the Post-Office at Philadelphia, and admitted for transmission through the mails at second-class rates.

**Subscriptions must begin with the January or July number.**

$2.50 per Annum, in Advance. Single Copies, 25 Cents.

PRINTED BY J. B. LIPPINCOTT COMPANY, PHILADELPHIA.

